# Quality of therapy and mental health among occupational therapists during the COVID-19 pandemic

**DOI:** 10.3389/fpubh.2022.1053703

**Published:** 2022-12-15

**Authors:** Ayahito Ito, Daisuke Sawamura, Shogo Kajimura, Hideki Miyaguchi, Haruki Nakamura, Toshiyuki Ishioka

**Affiliations:** ^1^Research Institute for Future Design, Kochi University of Technology, Kochi, Japan; ^2^Department of Psychology, University of Southampton, Southampton, United Kingdom; ^3^Faculty of Health Sciences, Hokkaido University, Sapporo, Japan; ^4^Faculty of Information and Human Sciences, Kyoto Institute of Technology, Kyoto, Japan; ^5^Department of Human Behavior Science of Occupational Therapy, Graduate School of Biomedical and Health Sciences, Hiroshima University, Hiroshima, Japan; ^6^Japanese Association of Occupational Therapists, Tokyo, Japan; ^7^Department of Occupational Therapy, Saitama Prefectural University, Saitama, Japan

**Keywords:** COVID-19, occupational therapy, anxiety, depression, insomnia

## Abstract

**Introduction:**

The coronavirus disease of 2019 (COVID-19) has had a severe psychological impact on occupational therapists. Clarifying the mental health status of occupational therapists and its relationship with therapy quality is essential for maintaining the quality of care and patients' quality of life. Therefore, the present study aimed to investigate whether and how mental health problems are related to the quality of occupational therapy.

**Methods:**

A nationwide cross-sectional online survey was conducted during Japan's second national state of emergency (January 2021). A total of 4,418 registered occupational therapists who were members of the Japanese Association of Occupational Therapists participated in this study. After screening for the exclusion criteria, data from 1,966 participants were analyzed.

**Results:**

Path analysis showed that insufficient information provision by the workplace and increased workload were associated with depression, anxiety, and insomnia. Specifically, depression was associated with decreased therapy quality. Furthermore, one's therapy quality showed a strong positive correlation with colleagues' therapy quality.

**Discussion:**

These results demonstrated a direct link between therapists' mental health conditions and therapy quality and suggested that decreased therapy quality might occur at the institutional rather than individual level. A reassessment of the support system and prompt detection and support for professionals with psychological symptoms may be the key to enhancing therapy quality and patients' quality of life. The present results contribute to the understanding of these relationships, considering the current pandemic context for occupational therapists.

## Introduction

The coronavirus disease 2019 (COVID-19) pandemic has had an unprecedented impact on society and is viewed as a global stressor induced by widespread voluntary restrictions and social distancing (1). The psychological effects of the COVID-19 outbreak on medical workers who have been fighting on the frontlines and on the general population have recently been documented (2, 3). The importance of physical and psychological support, such as the provision of precautionary items and information, has been emphasized (4). In this critical situation, medical workers who are directly or indirectly involved in diagnosis or treatment are at risk of developing psychological problems due to changes in workloads and/or work contents (3, 5).

Psychological impacts of COVID-19 on second-line healthcare professionals have been documented (5–8). Occupational therapists are classified as second-line medical professionals who do not directly care for patients with COVID-19 during the early stages (9). However, the work environment has changed due to the current pandemic, and this has had a negative impact on their mental health (6, 9). Although such psychological stress can negatively affect therapy quality and lead to client dissatisfaction (10), little is known about the relationship between mental health problems and the therapy quality of occupational therapists. Previous studies identified the relationship between mental health problems and job performance (11–19). For example, Shirom et al. (17) revealed that emotional exhaustion caused by overload is a critical factor in decreasing care quality, suggesting the importance of caring for the mental health of medical professionals to maintain the quality of care. Thus, the purpose of the present study was to clarify the psychological impact of the COVID-19 outbreak on occupational therapists and examine whether their mental health problems are related to decreased quality of therapy. By conducting a cross-sectional web-based survey targeted at registered occupational therapists in Japan, we investigated the relationships among the changes in work and life due to COVID-19, mental health problems, and quality of therapy. As mentioned below, we focused on the following four factors that can affect mental health conditions: efforts to avoid being infected, information provision from the workplace, workload, and working hours.

Previous studies investigating the effects of quarantine on mental health conditions have documented that healthcare workers, compared with the general public, exhibited concerns about being infected by others and reported substantially more negative feelings, such as anger and loneliness, after quarantine (20). A previous report showed that 98.3% of occupational therapists showed more significant efforts to avoid infection, and 94.7% of them refrained from unnecessary outings (6). As a decrease in social connectedness is related to perceived stress (1), these results raise the possibility that efforts to avoid being infected would be related to adverse psychological effects. Information provision from the workplace also has a critical role in addressing mental health conditions. A recent report suggested that sufficient information from the workplace significantly reduced the risk of mental health problems (6, 21), and this finding is consistent with previous evidence that showed the effectiveness of information provision for mental support during previous infectious outbreaks such as H1N1 and SARS (22–24). Other recent reports suggested that social connectedness was associated with a lower level of perceived stress and COVID-19-related burnout (1, 25). Thus, the information provided may improve mental health conditions by enhancing the sense of social connectedness.

The increased workload and working hours negatively impact mental health conditions (6, 26–30). For example, previous literature showed that workload is positively related to depression and that its relationship was mediated by emotional distress (29), and long working hours are also associated with depression (31) and further associated with poor patient safety and decreased care quality (30). In addition to this physical overload, the present pandemic forces medical professionals to work in high-pressure environments (26).

**Figure 1:**
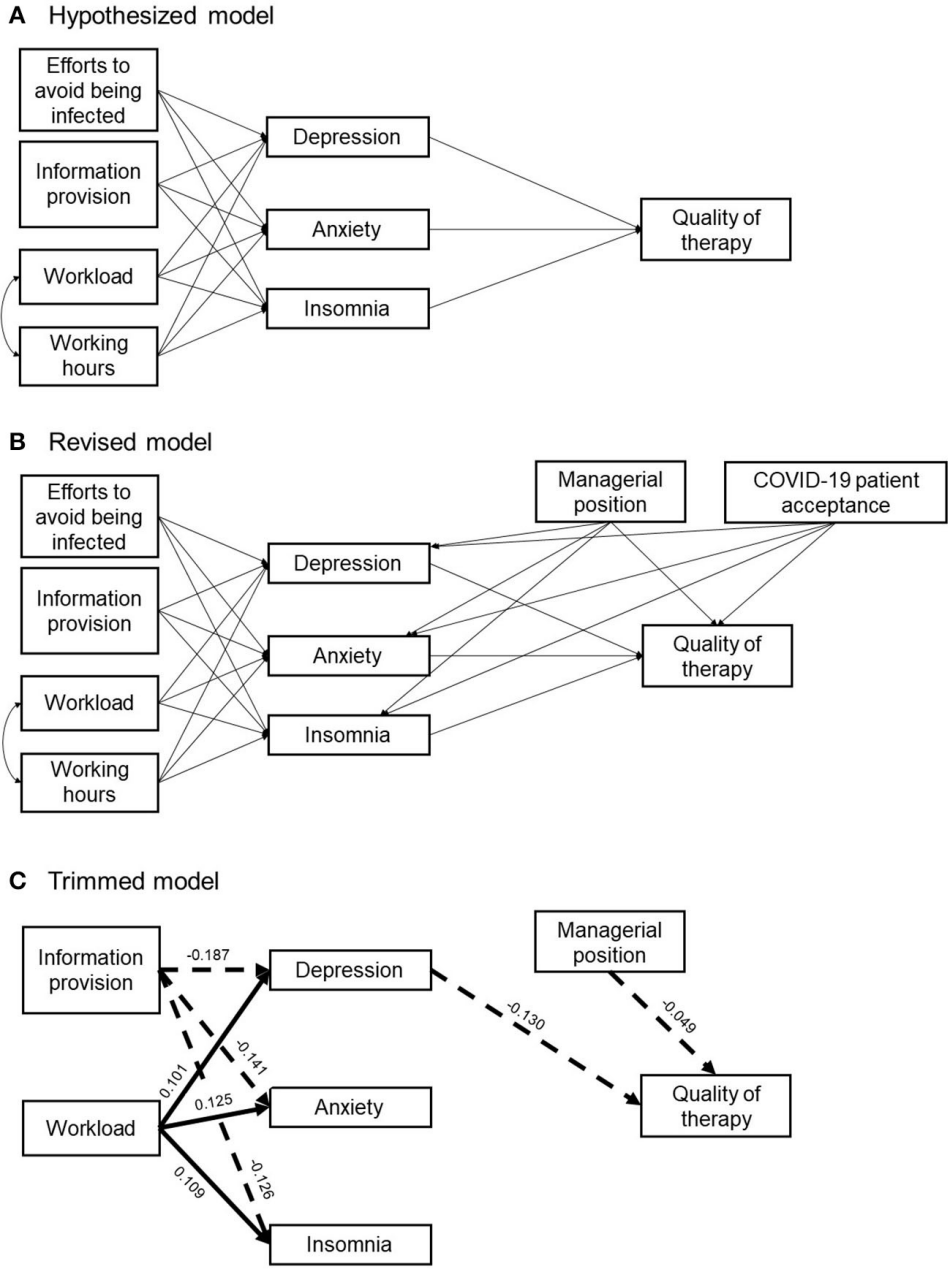
A hypothesized path model **(A)**, revised model **(B)**, and trimmed model **(C)**. In the trimmed model, the straight line depicts a significantly positive relationship, and the dashed line depicts a significantly negative relationship.

To replicate these findings (i.e., the link between environmental factors and mental health) and further examine whether these mental health problems negatively affect the quality of therapy, we used path analysis in the present study. Because of the close relationships among mental health problems, including depression, anxiety, and insomnia (32, 33), in our hypothesized model, we assume that the four factors (i.e., efforts to avoid being infected, information provision from the workplace, workload, and working hours) are linked to each psychological symptom, and all three psychological symptoms would be related to therapy quality. Based on previous findings, which showed relationships among workload, mental health, and job performance (11, 17, 18), we designed a hypothesized path model (Figure 1A). This model focused on the relationships between (1) the changes in work and life due to COVID-19, (2) mental health problems, including depression, anxiety, and insomnia, and (3) the quality of therapy. Based on recent literature that has documented (perceived) social isolation (20, 23), we proposed “efforts to avoid being infected” and “less information provision from the workplace” as candidates that may increase mental health problems. Second, based on literature that focused on the relationship between mental health and job performance (11, 17, 18, 27, 30), we hypothesized that the increase in mental health problems would be related to a decrease in therapy quality. Based on previous reports that showed a close link between perceptions of caregiver and client (34, 35), in the present study, we employed self-report of one's therapy quality as an index of therapy quality. Further, to examine whether one's therapy quality is closely related to colleagues' therapy quality (i.e., to determine if the change in quality is beyond personal problems), we performed a simple correlation analysis.

## Materials and methods

### Research protocol

This cross-sectional online survey was conducted in Japan from 20 to 25 January 2021. The data were collected through Google Forms (https://www.google.com/forms/about/). All the respondents were registered occupational therapists who were members of the Japanese Association of Occupational Therapists. A request for participation was sent to all registered members *via* email on 20 January 2021. The study protocol was approved by the ethical committee at Saitama Prefectural University (acceptance number: 20003). All participants provided written, informed consent. Email addresses were collected to ensure that the same respondent did not take the questionnaire multiple times.

### Online questionnaire

Participants were asked to report their sociodemographic characteristics: age, gender, academic background, marital status (married or unmarried), history of psychiatric disorders (yes or no), employment type (full-time/part-time), managerial position (yes or no), and years of service. As the participants had to answer each question before proceeding, no missing data existed. Participants who reported a history of psychiatric disorders were excluded from the analysis. Based on previous findings (5, 6, 23), we focused on loneliness, depression, anxiety, and insomnia. Three validated questionnaires were used: the Japanese version of the three-item loneliness scale (36), the Zung Self-Rating Anxiety Scale (SAS) (37), the Zung Self-Rating Depression Scale (SDS) (38), and the Japanese version of the Insomnia Severity Index (ISI-J) (39). The three-item loneliness scale measures overall with three items (“I feel that I lack companionship,” “I feel left out,” and “I feel isolated from others”) and is known to identify loneliness quite well (40). The total score ranged from 3 to 9, and higher scores reflected greater loneliness (40). The SAS and SDS had 20 items each that measured anxiety and depression, respectively. The SAS included negative statements such as “I get upset easily or feel panicky” (37). The SDS contained 10 negative statements, such as “I feel down-hearted and blue,” and 10 reverse-scored positive statements, such as “My life is pretty full” (38). The ISI-J contained seven questions assessing the nature, severity, and impact of insomnia, rated on a five-point Likert scale (0 = no problem; 4 = very severe problem) (6, 41). These questionnaires have been widely used for non-clinical samples, and the cutoffs for detecting the presence or absence of loneliness, anxiety, depression, and insomnia were ≥ 6 for the three-item loneliness scale (42–44), ≥ 40 for the SAS (45), ≥ 50 for the SDS (46), and ≥ 10 for the ISI-J (39, 47).

Participants were also asked to answer the following items concerning the work environment: acceptance of patients with COVID-19 at their workplace (yes or no), provision of information on COVID-19 by the workplace (7-point rating scale ranging from 1 = *insufficient* to 7 = *sufficient*), changes in one's therapy quality compared to the period before COVID-19 (decreased, unchanged, or increased), colleagues' therapy quality compared with the period before COVID-19 (worse, unchanged, or better), changes in working hours compared to the period before COVID-19 (increase, decrease, or no change), changes in workload with the period before COVID-19 (increase, decrease, or no change), work from home (yes or no), and free description (fill-in-the-blank question).

Concerning daily life, participants were asked to respond to the following items concerning everyday life: efforts to avoid being infected (7-point rating scale ranging from 1 = *never* to 7 = *frequent*), efforts to not transmit the virus to others (7-point rating scale), frequency of contact with family (7-point rating), frequency of contact with friends (7-point rating), changes in daily step count compared to the period before COVID-19 (which was evaluated using records automatically logged in healthcare applications implemented in the respondents' phones), fewer outings (yes or no), attempts to avoid face-to-face conversations (yes or no), increased standard precautions at home (handwashing and gargling) (yes or no), increased frequency of mask-wearing (yes or no), increased social networking site usage (yes or no), and free description (fill-in-the-blank question).

### Path model

Based on previous findings, which showed relationships among workload, mental health, and job performance (11, 17, 18), we designed a hypothesized path model (Figure 1A). This model focused on the relationships between the changes in work and life due to COVID-19, mental health problems including depression, anxiety, and insomnia, and the quality of therapy. Based on recent literature that has documented (perceived) social isolation and loneliness (20, 23), we chose “efforts to avoid being infected” and “less information provision from the workplace” as candidates that can exacerbate mental health problems. Second, based on literature that focused on the relationship between mental health and job performance (11, 17, 18, 27, 30), we hypothesized that the exacerbation of mental health problems would be related to a decrease in therapy quality. Based on previous reports that showed a close link between the perceptions of caregiver and client (34, 35), in the present study, we employed self-report of one's therapy quality as an index of therapy quality. Further, to examine whether one's therapy quality was closely related to colleagues' therapy quality (i.e., check if the change in quality is beyond personal problems), we performed a simple correlation analysis. Path analyses were performed using AMOS 28 (48). To assess the goodness of fit, we employed the chi-square value, the comparative fit index (CFI), and the root-mean-squared error of approximation (RMSEA). The chi-square value of < 0.05, the CFI value of ≥ 0.95, and the RMSEA value of < 0.06 are considered to indicate good model fit (49–52). The data were evaluated for estimation methods that assume multivariate normality using Bollen-Stine bootstrapping. Skewness and kurtosis were also examined (Supplementary Table 1).

## Results

### Sample characteristics and questionnaire results

Sample characteristics and questionnaire results are shown in Table 1. Cutoff scores for the four questionnaires were determined based on previous literature (39, 42, 45, 46). The total number of respondents was 4,418. Data from respondents with a history of psychiatric disorders (*n* = 481), inconsistent answers between yes or no questions and rating (e.g., “yes” to the change in outing frequency but rated the frequency as unchanged) (*n* = 1,336), a declaration that they do not actively see clients (*n* = 475), and inconsistent answers to working hours (*n* = 160) were excluded. The remaining respondents were 1,966 (1,106 women and 860 men).

**Table 1:** Sample characteristics and questionnaire results.

**Survey item**	**No./total No. (%)**
	**All 47 Prefectures** **(*n* = 1,966)**
**Sample characteristics**	
Age, M (SD)	36.8 (8.8)
**Gender**	
Women	1,106 (56.3)
Men	860 (43.7)
**Academic background**	
<Bachelor's	1,115 (56.7)
≧ Bachelor's	851(43.3)
**Marital status**	
Married	1,229 (62.5)
Unmarried	737 (37.5)
**Employment type**	
Full time	1,875 (95.4)
Part time	91 (4.6)
**Managerial position**	
Yes	651 (33.1)
No	1,315 (66.9)
Service years, M (SD)	12.8 (8.0)
**Questionnaire results**	
**Presence of anxiety, depression, insomnia, and loneliness (cutoff score)**	
Loneliness (≧6)	480 (24.4)
SDS (≧50)	325 (16.5)
SAS (≧40)	297 (15.1)
ISI (≧10)	286 (14.5)
**Median score on each questionnaire (IQR)**	
Loneliness	4 (3–5)
SDS	40 (34–47)
SAS	33 (29–37)
ISI	5 (2–8)
**Accepting patients with COVID-19**	
Yes	536 (27.3)
No	1,430 (72.7)
**Items related to work**	
**Provision of information on COVID-19 by workplace (1 = never, 7 = sufficient)**	
5–7 (above average level)	1,463 (74.4)
1–3 (below average level)	185 (9.4)
4 (average)	318 (16.2)
**Changes in one's therapy quality compared with early 2019 (before COVID-19)**	
Increased	123 (6.3)
Decreased	423 (21.5)
Unchanged	1,420 (72.2)
**Changes in colleagues' therapy quality compared with early 2019 (before COVID-19)**	
Increased	110 (5.6)
Decreased	399 (20.3)
Unchanged	1,457 (74.1)
**Changes in working hours compared with early 2019 (before COVID-19)**	
Increased	165 (8.4)
Decreased	173 (8.8)
Unchanged	1,628 (82.8)
**Changes in workload compared with early 2019 (before COVID-19)**	
Increased	990 (50.4)
Decreased	336 (17.0)
Unchanged	640 (32.6)
**Work from home**	
Yes	147 (7.5)
No	1,819 (92.5)
**Free description about changes in work style (fill-in-the-blank question)**	
Yes	345 (17.5)
No	1,621 (82.5)
**Items related to private life**	
**Efforts to avoid being infected (1 = *never*, 7 = *frequent*)**	
5–7	1,951 (99.2)
1–3	1 (0.05)
4	14 (0.7)
**Efforts to not transmit the virus to others (1 = *never*, 7 = *frequent*)**	
5–7	1,943 (98.8)
1–3	6 (0.3)
4	17 (0.9)
**Frequency of contact with family (1 = *never*, 7 = *frequent*)**	
5–7	1,342 (68.3)
1–3	260 (13.2)
4	364 (18.5)
**Frequency of contact with friends (1 = *never*, 7 = *frequent*)**	
5–7	499 (25.4)
1–3	942 (47.9)
4	525 (26.7)
**Changes in daily step count compared with early 2019**	
Increased	163 (8.3)
Decreased	339 (17.2)
Unchanged	1,386 (70.5)
Unknown	78 (4.0)
**Fewer outings**	
Yes	1,938 (98.6)
No	28 (1.4)
**Avoidance of face-to-face conversations**	
Yes	1,808 (92.0)
No	158 (8.0)
**Increased precautions at home**	
Yes	1,893 (96.3)
No	73 (3.7)
**Increased mask-wearing**	
Yes	1,954 (99.4)
No	12 (0.6)
**Increased SNS usage**	
Yes	1,003 (51.0)
No	963 (49.0)
**Free description about changes in life (fill-in-the-blank question)**	
Yes	329 (16.7)
No	1,637 (83.3)

SAS, Self-rating Anxiety Scale, SDS, Self-rating Depression Scale, ISI, Insomnia Severity Index, IQR, interquartile range, COVID-19, coronavirus disease 2019. Percentages may not total 100 because of rounding.

The results of bivariate correlations among study variables are shown in Table 2. Information provision is negatively associated with the three psychological symptoms, supporting previous evidence that insufficient information provision is related to mental health problems. Information provision and the three psychological symptoms were significantly related to one's quality of therapy (all *p*-values < 0.01). In other words, an increase in information provision is positively related to the quality of therapy, whereas a decrease in psychological symptoms is positively related to the quality of therapy. Efforts to avoid being infected, workload, and working hours did not show a significant relationship with the quality of therapy.

**Table 2:** Correlations among study variables.

**Variable**	**1**	**2**	**3**	**4**	**5**	**6**	**7**
1. Care not to be infected							
2. Information provision	0.22^***^						
3. Workload	0.07^**^	0.04					
4. Working hours	−0.004	−0.01	0.27^***^				
5. Depression	−0.03	−0.18^***^	0.09^***^	0.01			
6. Anxiety	0.003	−0.13^***^	0.12^***^	0.04	0.73^***^		
7. Insomnia	−0.03	−0.12^***^	0.10^***^	0.04	0.52^***^	0.53^***^	
8. Quality of therapy	0.01	0.08^***^	0.02	0.02	−0.13^***^	−0.10^***^	−0.06^**^

*** *p* < 0.001,

** *p* < 0.01,

* < 0.05. Absolute values of correlation coefficients >0.1 are shown in bold.

Furthermore, efforts to avoid being infected and working hours were not significantly associated with any psychological symptoms. We also performed correlation analysis using the data of one's therapy quality and colleagues' therapy quality. This analysis showed a strong positive correlation (Pearson's r = 0.79, *p* < 0.01, 95% confidence interval [0.78, 0.81]), suggesting that changes in therapy quality during the pandemic mainly occur at the institutional rather than the individual level.

### Path analysis

First, we examined whether the demographic variables, including gender, managerial position, marital status, and acceptance of patients with COVID-19, needed to be considered as control variables using multigroup analysis. The four types of multigroup analysis revealed that managerial position and acceptance of patients with COVID-19 needed to be considered as control variables, whereas gender and marital status did not make significant group differences (Supplementary Figures 1–8). Therefore, we revised the model to include managerial position and acceptance of patients with COVID-19 as control variables and performed the path analysis (Figure 1B). The path analysis for the revised model (Table 3, Figure 1B) showed a significant chi-square value (χ2 = 117.22, df = 9, *p* < 0.001) and a discrepancy between the model and data (Bollen-Stine bootstrapping, *p* < 0.05), but the other goodness-of-fit indicators showed that this model had a good fit (CFI = 0.96, RMSEA = 0.078). Next, we designed a trimmed model based on the results of the revised model. In this trimmed model, exogenous variables, “efforts to avoid being infected” and “working hours,” and a control variable, “acceptance of patients with COVID-19,” which was not related to any other variables and had insignificant paths, were removed (Figure 1C). Although, this model showed a significant chi-square value (χ2 = 42.48, df = 7, *p* < 0.05), and the discrepancy between the model and data (Bollen-Stine bootstrapping, *p* < 0.05), the other goodness-of-fit indices showed that this model had a better fit (CFI = 0.99, RMSEA = 0.05). These results suggested that insufficient information provision by the workplace and that increased workload are critically associated with mental health problems, and therapists in managerial positions tend to feel that the quality of their therapy has decreased during the COVID-19 pandemic. Furthermore, although the bivariate correlation revealed that all mental health problems were significantly associated with the quality of therapy, depression may be especially important in maintaining the quality of therapy.

**Table 3:** Path coefficients of the revised model and trimmed model.

	**Unstandardized estimate**	**Standard error**	***p*-value**	**Standardized estimate**
**Revised model**				
Care not to be infected → Depression	0.006	0.296	0.984	0.000
Care not to be infected → Anxiety	0.265	0.222	0.234	0.026
Care not to be infected → Insomnia	−0.04	0.136	0.772	−0.006
Information provision → Depression	−1,208	0.151	<0.001^***^	−0.176
Information provision → Anxiety	−0.705	0.113	<0.001^***^	−0.138
Information provision → Insomnia	−0.374	0.069	<0.001^***^	−0.121
Workload → Depression	0.843	0.163	<0.001^***^	0.119
Workload → Anxiety	0.689	0.122	<0.001^***^	0.131
Workload → Insomnia	0.352	0.075	<0.001^***^	0.110
Working hours → Depression	0.005	0.021	0.806	0.006
Working hours → Anxiety	−0.03	0.045	0.513	−0.015
Working hours → Insomnia	0.005	0.034	0.888	0.003
Depression → Quality of care	−0.007	0.002	<0.001^***^	−0.115
Anxiety → Quality of care	−0.002	0.003	0.429	−0.027
Insomnia → Quality of care	0.001	0.003	0.791	0.007
Managerial position → Depression	−2,103	0.41	<0.001^***^	−0.114
Managerial position → Anxiety	−0.804	0.307	0.009^*^	−0.059
Managerial position → Insomnia	−0.346	0.188	0.066	−0.041
Managerial position → Quality of care	−0.053	0.024	0.028*	−0.049
Acceptance of COVID-19 patient → Depression	0.038	0.429	0.93	0.002
Acceptance of COVID-19 patient → Anxiety	−0.206	0.322	0.522	−0.014
Acceptance of COVID-19 patient → Insomnia	−0.305	0.197	0.122	−0.035
Acceptance of COVID-19 patient → Quality of care	−0.047	0.025	0.065	−0.041
**Trimmed model**				
Information provision → Depression	−1.283	0.151	<0.001^***^	−0.187
Information provision → Anxiety	−0.718	0.113	<0.001^***^	−0.141
Information provision → Insomnia	−0.393	0.069	<0.001^***^	−0.126
Workload → Depression	0.716	0.156	<0.001^***^	0.101
Workload → Anxiety	0.657	0.117	<0.001^***^	0.125
Workload → Insomnia	0.350	0.071	<0.001^***^	0.109
Depression → Quality of care	−0.008	0.001	<0.001^***^	−0.130
Managerial position → Anxiety	0.295	0.206	0.153	0.021
Managerial position → Quality of care	−0.053	0.024	0.028*	−0.049

*** *p* < 0.001,

^*^ < 0.05. Significant *p*-values are shown in bold.

## Discussion

Using data from an online questionnaire from registered occupational therapists, we investigated the relationships among work life, mental health conditions, and quality of therapy. The results from the path analysis showed that insufficient information provision at the workplace and increased workload were related to depression, anxiety, and insomnia. Depression was associated with decreased self-evaluation of one's quality of therapy. Furthermore, the evaluation of one's quality of therapy showed a strong positive correlation with the evaluation of a colleague's quality of therapy, suggesting the possibility that changes in therapy quality occur on an institutional basis rather than on an individual basis. However, the majority of participants thought that they obtained information from the workplace at a higher level than average; it is considered that qualitatively novel supporting systems and reassessment of workload are important.

A total of 50.4% of the respondents reported an increased workload due to the pandemic, and the increase in workload was significantly related to an increase in depression, anxiety, and insomnia. These findings are consistent with a recent meta-analysis focusing on burnout and the mental health of medical professionals (53). The path analysis showed a specific link between depression and therapy quality. Although depression showed a strong positive correlation with the other two symptoms (*r* = 0.73 with anxiety and *r* = 0.52 with insomnia), anxiety and insomnia showed relatively smaller correlations with therapy quality, which could be negligible (*r* = −0.1 for anxiety and *r* = −0.06 for insomnia) (54). Depression showed a relatively larger correlation with therapy quality (*r* = −0.13), highlighting the relationship between depression and the quality of therapy. Contrary to our hypothesis, efforts to avoid being infected did not show a significant link with other variables and were excluded from our trimmed model. This may be because there is an extremely high number of therapists who do not want to be infected. In fact, 99.2% of the respondents said that their efforts to avoid being infected were above average. The data might indicate the professionalism of occupational therapists.

During the second state of emergency, 24.4, 16.5, 15.1, and 14.5% of occupational therapists presented symptoms of loneliness, depression, anxiety, and insomnia, respectively. Although the prevalence of psychological symptoms varies across countries, especially due to the pandemic (55), the ratio of respondents who showed depression and anxiety increased, and the ratio of insomnia decreased in comparison with the previous online survey, which was conducted during the initial state of emergency (10.9 to 16.5% for depression, 11.3 to 15.1% for anxiety, and 16.8 to 14.5% for insomnia) (6). Although the number of people with insomnia decreased, mental health condition among therapists seems to be getting worse, and additional mental support is needed. Taken together with the finding of the bivariate correlation, which showed a negative relationship between the increase in psychological symptoms and therapy quality, there is a possibility that therapy quality has decreased throughout the pandemic in some institutions. Although additional direct and causal evidence is needed, insufficient information provision and overload might negatively affect therapy quality, resulting in decreased quality of life of patients.

It should be noted that 74.4% of respondents answered that the information provided was above average, and only 9.4% answered that the information provided was below average. Thus, most workplaces seem to have been trying to support therapists, and therapists acknowledge the effort. However, in addition to typical support for workers, such as providing personal protective equipment and information, individual psychological support would be needed. For example, one possible way is to identify therapists with psychological symptoms using validated questionnaires (9) and monitor and care for them (26). Burnout among healthcare professionals has been a severe problem for a long time (10, 30), and it is becoming more serious in these challenging times. As burnout is linked to decreased therapy quality, such an approach is essential for therapists and patients.

Another possible idea to support therapists can be drawn from a recent randomized clinical trial that demonstrated the effectiveness of a layperson-delivered, empathy-focused program (56). In this study, callers who were briefly trained in empathetic conversational techniques using a 1-h videoconference talked to participants over the telephone for 4 weeks, and this intervention improved depression, loneliness, and anxiety in at-risk adults (56). If managers are trained to have empathetic conversations using short material, such an intervention can be implemented in each workplace, and it would have the potential to support therapists and further maintain therapy quality during pandemics. However, it should be noted that the managerial position has a negative relationship with therapy quality, and care for the managers is also essential. The findings of this study revealed that one's therapy quality showed a strong positive correlation with colleagues' therapy quality. Thus, it is plausible that therapy quality can change at the institutional level. As a first step, it is crucial to reassess the work and mental health conditions of each professional and the supporting system in each institution. Then, it might be helpful to consider employing such additional approaches.

The current findings have limitations. First, as the present results were based on a cross-sectional online questionnaire and data were gathered only from occupational therapists in Japan, further studies are needed to examine whether these results can be applied to other populations (e.g., physiotherapists) and across borders. Second, the evaluation of one's own and colleagues' therapy quality in the present study was based on the participants' self-reports. Thus, there is a possibility that those self-evaluations are biased to some extent based on social desirability (a tendency to present oneself as socially desirable or acceptable rather than to present one's true thoughts or feelings) or by decreased self-esteem, which might be related to a personal mental health condition. Although the evaluation between one's therapy quality and that of colleagues' were correlated, and it is plausible that a decrease in therapy quality happens to some extent, these results are reported with caution. Future studies are needed to collect patient data relating to the quality of therapy and personality assessment, which may yield greater objectivity than self-reports. Finally, and most importantly, studies are required that directly examine whether interventions to support therapists' mental health, such as an empathy-focused program, are effective in terms of improving the quality of therapy and patients' quality of life. Nevertheless, we believe the findings from this study would be the cornerstone of a novel support system for therapists.

In conclusion, a nationwide cross-sectional online survey was conducted to investigate whether and how mental health problems are related to the therapy quality of occupational therapists. The results showed that insufficient information provision at the workplace and increased workload were positively related to psychological symptoms. Only depression was associated with decreased self-evaluation of one's quality of therapy. Evaluations of one's own quality and a colleague's quality of therapy showed a strong positive correlation, suggesting that improvements in therapy quality may occur at the institutional rather than the individual level.

## Data availability statement

The raw data supporting the conclusions of this article will be made available by the authors, without undue reservation.

## Ethics statement

The studies involving human participants were reviewed and approved by the Ethical Committee in Saitama Prefectural University. The patients/participants provided their written informed consent to participate in this study.

## Author contributions

AI, DS, HM, HN, and TI: study conception, design, and data acquisition. AI, DS, SK, and TI: analysis, interpretation of data, and writing—review and editing. AI: writing—original draft. All authors approved final version of the article.
