# Supplementary material for: Quality of therapy and mental health among occupational therapists during the COVID-19 pandemic
Source: Front Public Health. 2022 Dec 15;10:1053703. doi: 10.3389/fpubh.2022.1053703 (PMC9799253; doi:10.3389/fpubh.2022.1053703)
Supplement: Supplementary file 1 [file Data_Sheet_1.PDF]

Supplementary Figures

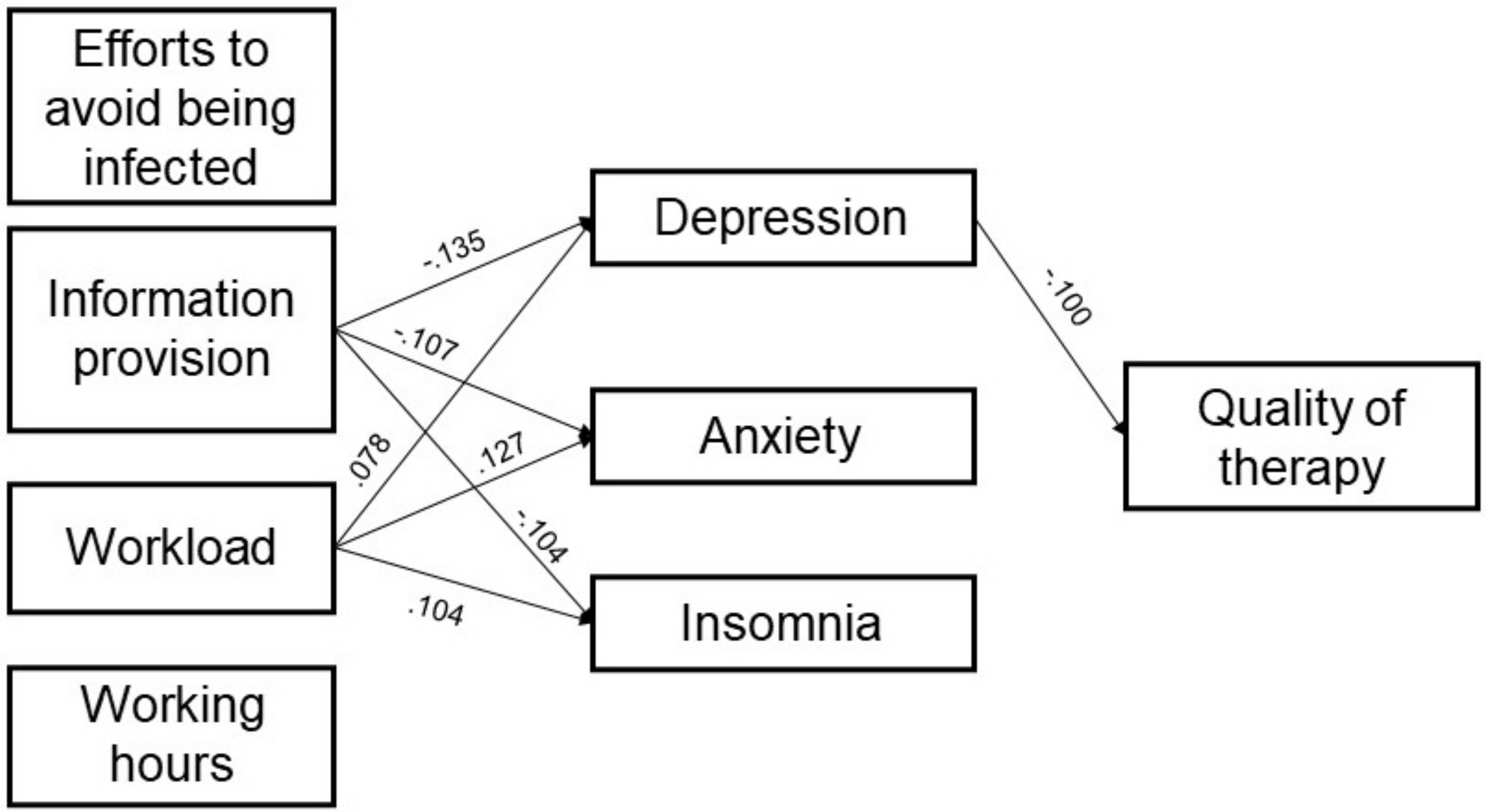

Supplementary Figure 1

Results of the path model for female participants (a multigroup analysis based on gender). Significant paths are depicted with coefficients. For visualization purpose, we retain variables which does not show any significant effects. The results showed no group differences (Supplementary Figure 2).

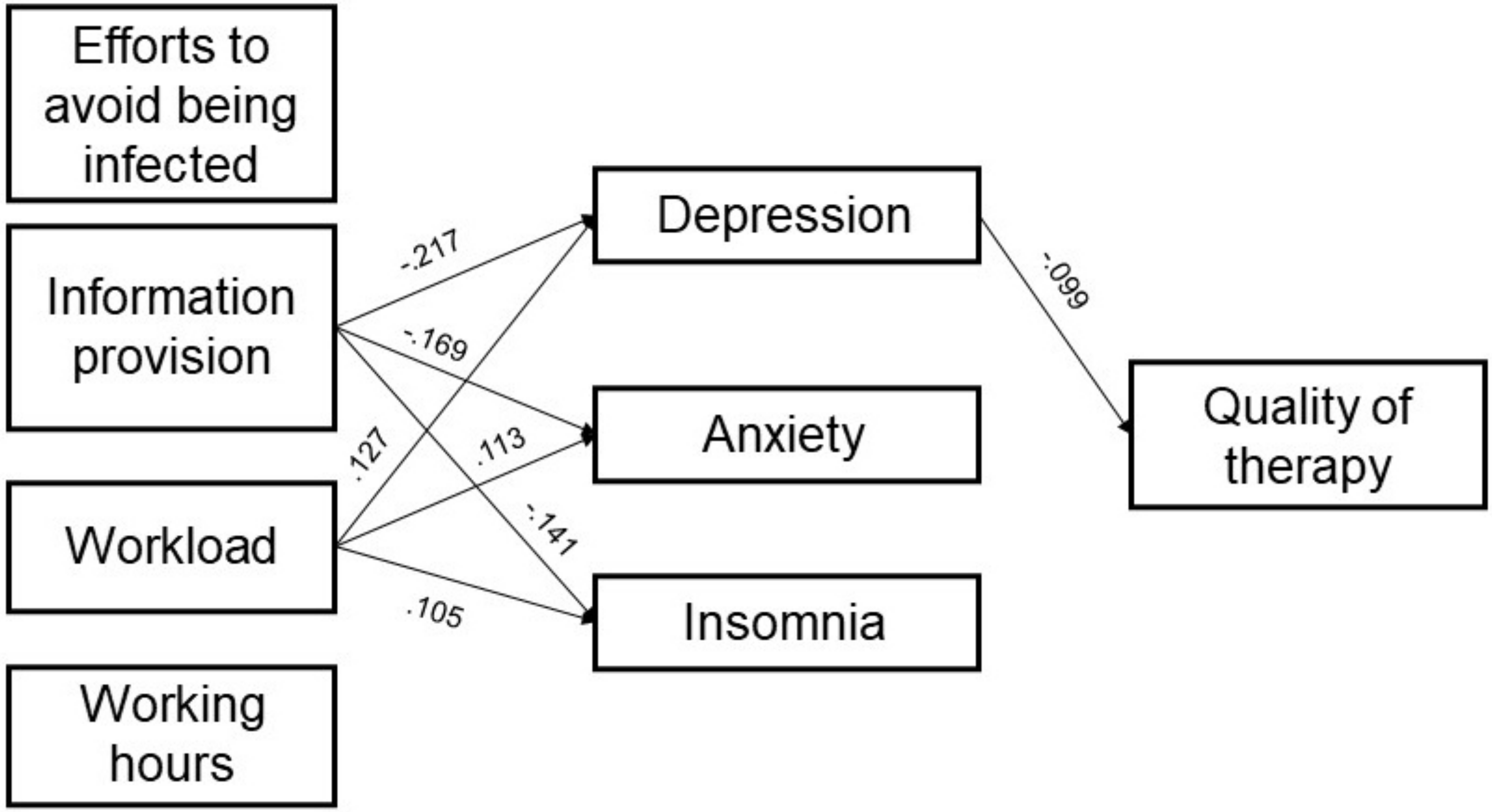

Supplementary Figure 2

Results of the path model for male participants (a multigroup analysis based on gender). Significant paths are depicted with coefficients. For visualization purpose, we retain variables which does not show any significant effects. The results showed no group differences (Supplementary Figure 1).

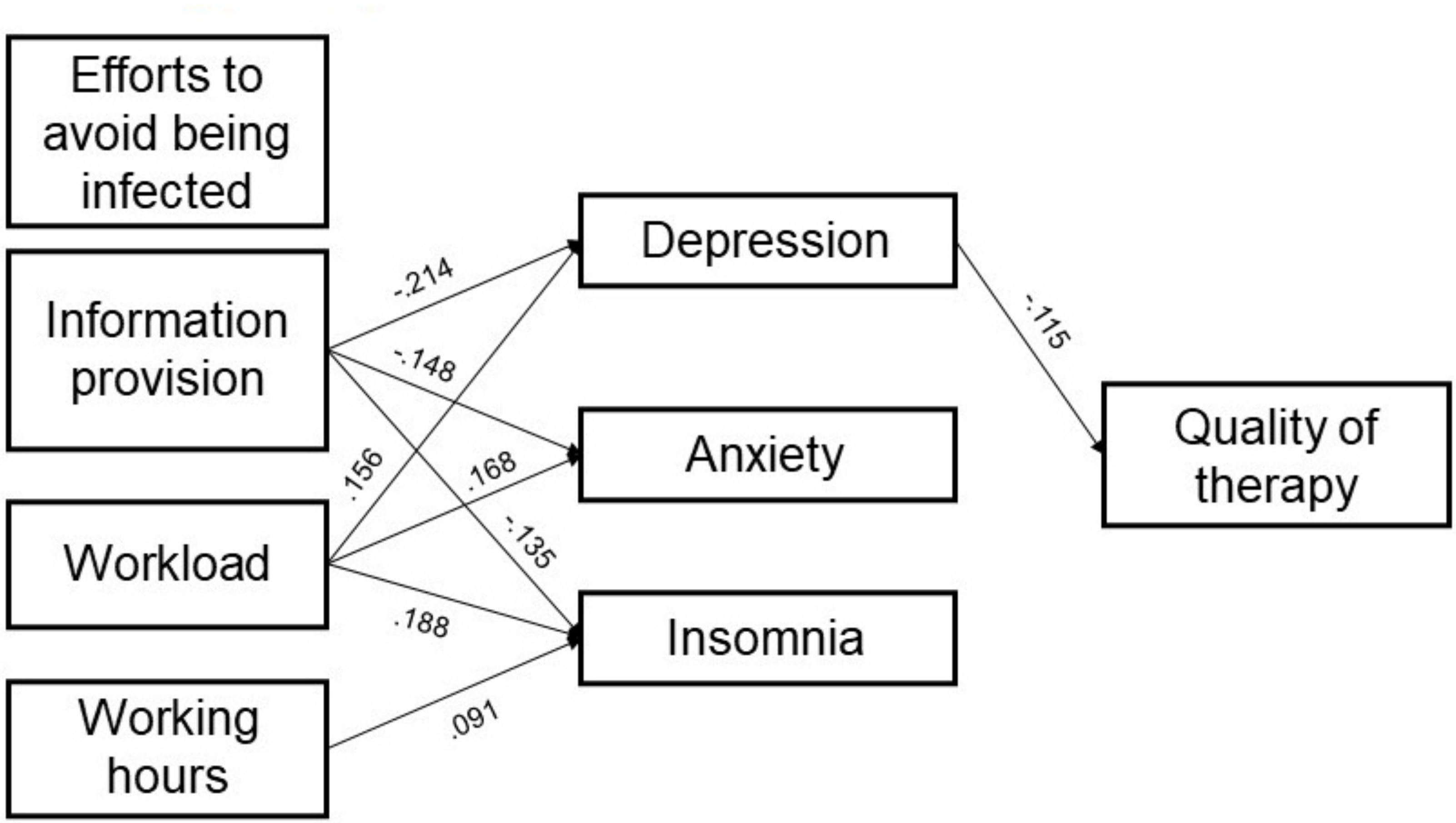

Supplementary Figure 3

Results of the path model for participants in managerial positions (A multigroup analysis based on managerial position). Significant paths are depicted with coefficients. For visualization purpose, we retain variables which does not show any significant effects. The results showed group differences in the link between "working hours" and insomnia (Supplementary Figure 4).

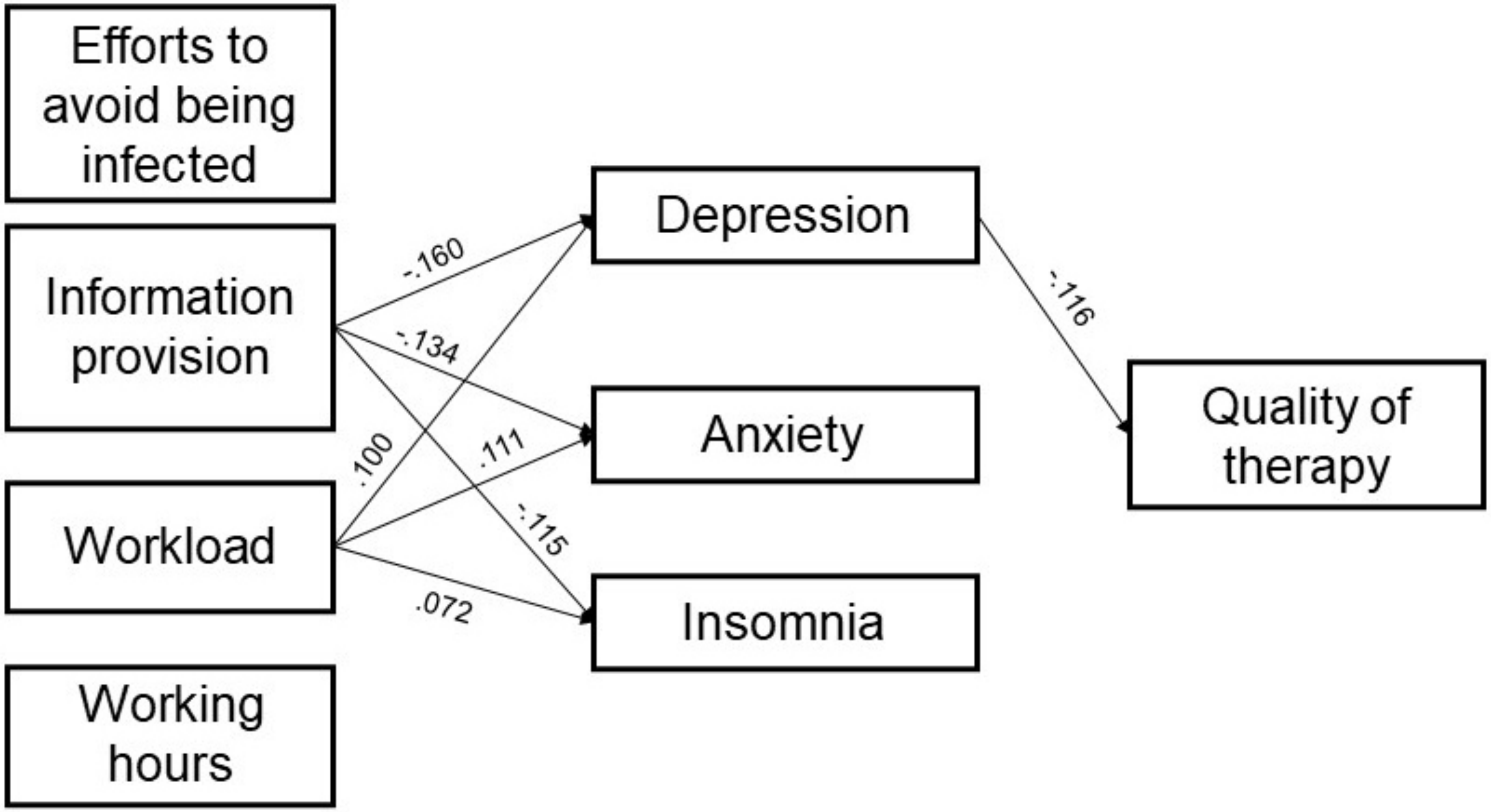

Supplementary Figure 4

Results of the path model for participants who are not in managerial positions (A multigroup analysis based on managerial position). Significant paths are depicted with coefficients. For visualization purpose, we retain variables which does not show any significant effects. The results showed group differences in the link between "working hours" and insomnia (Supplementary Figure 3).

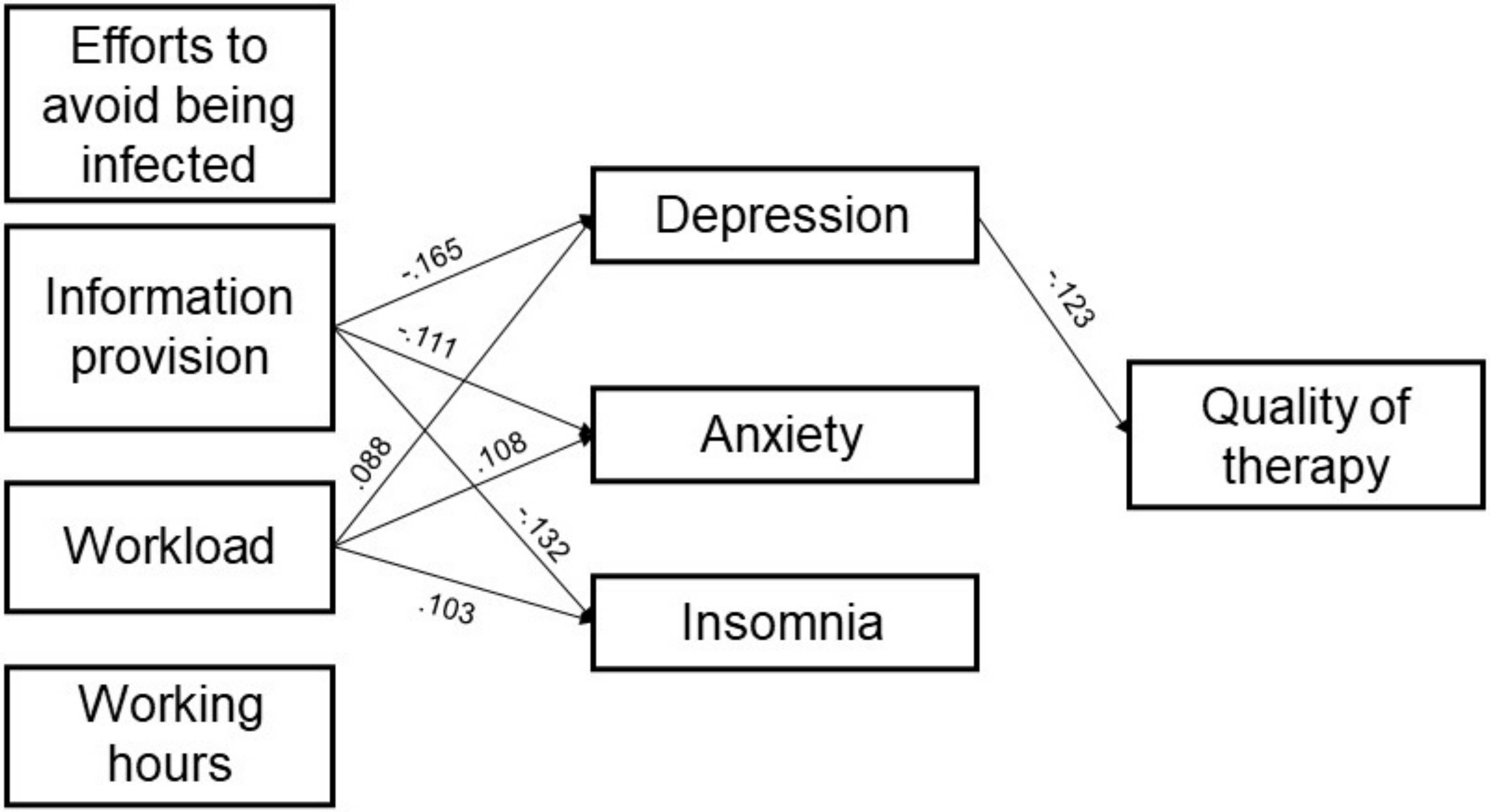

Supplementary Figure 5

Results of the path model for married participants (a multigroup analysis based on marriage). Significant paths are depicted with coefficients. For visualization purpose, we retain variables which does not show any significant effects. The results showed no group differences (Supplementary Figure 6).

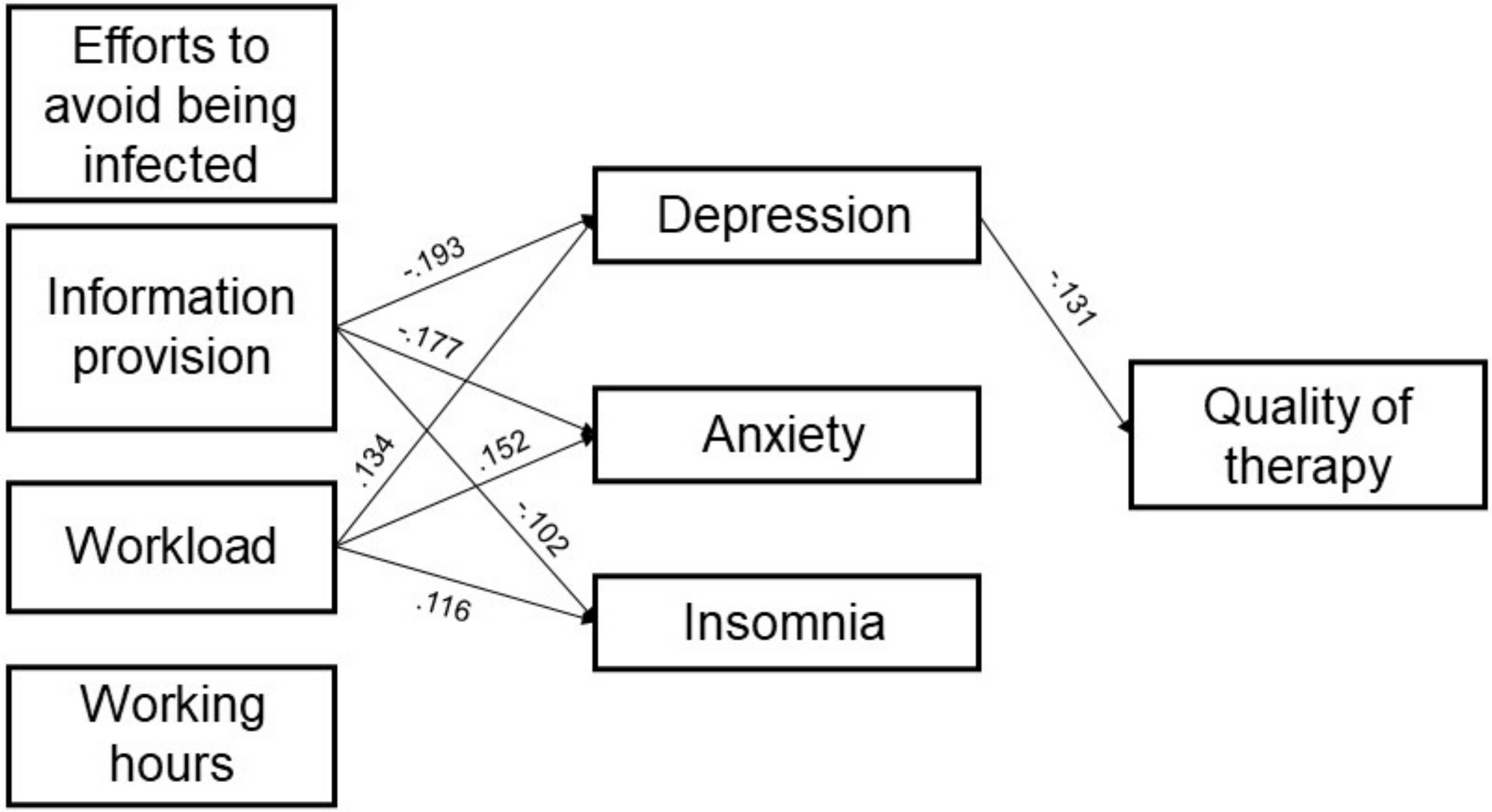

Supplementary Figure 6

Results of the path model for unmarried participants (a multigroup analysis based on marriage). Significant paths are depicted with coefficients. For visualization purpose, we retain variables which does not show any significant effects. The results showed no group differences (Supplementary Figure 5).

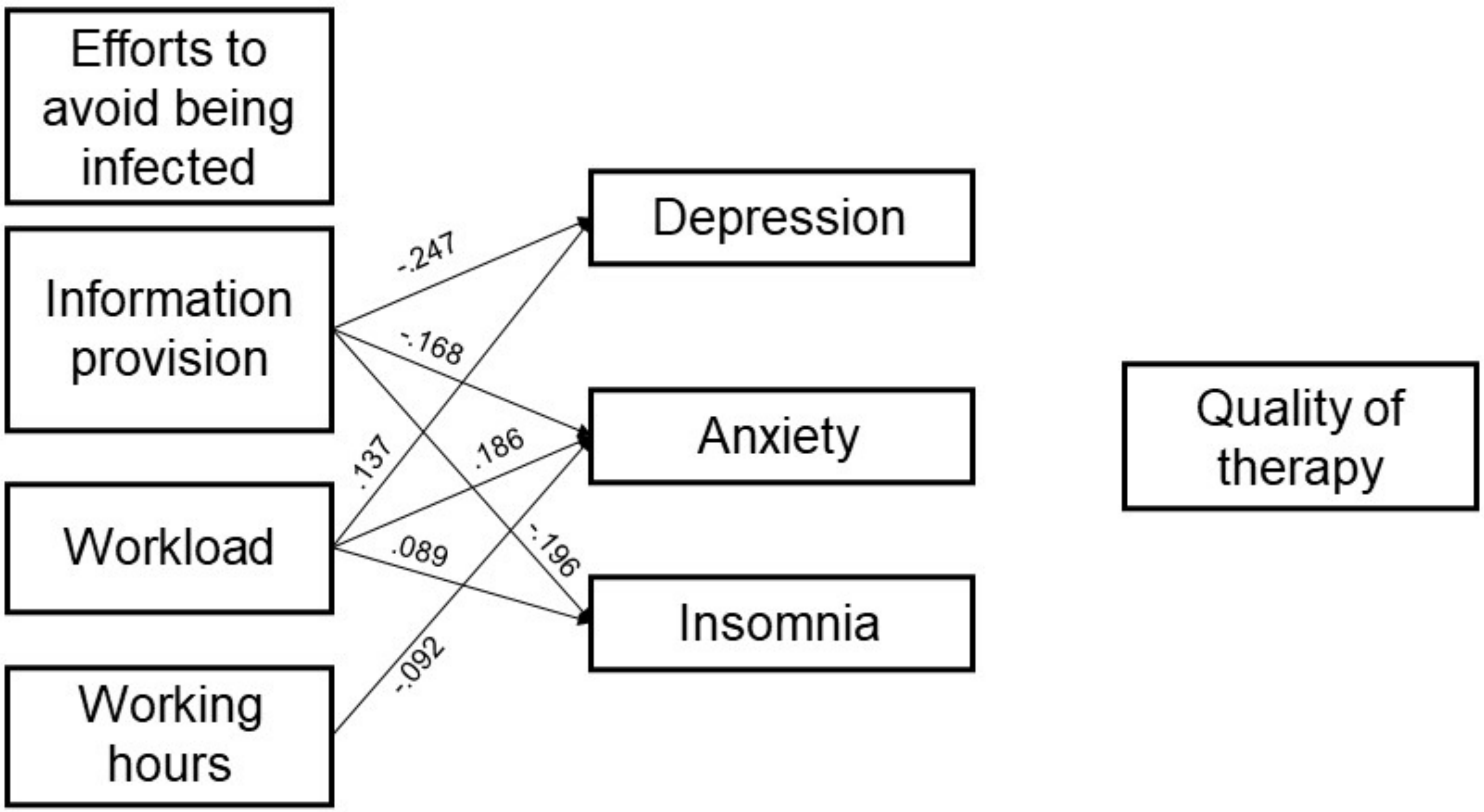

Supplementary Figure 7

Results of the path model for participants who works at hospitals that accept patients with COVID-19. Significant paths are depicted with coefficients. For visualization purpose, we retain variables which does not show any significant effects. The results showed group differences in the link between "working hours" and anxiety (Supplementary Figure 8).

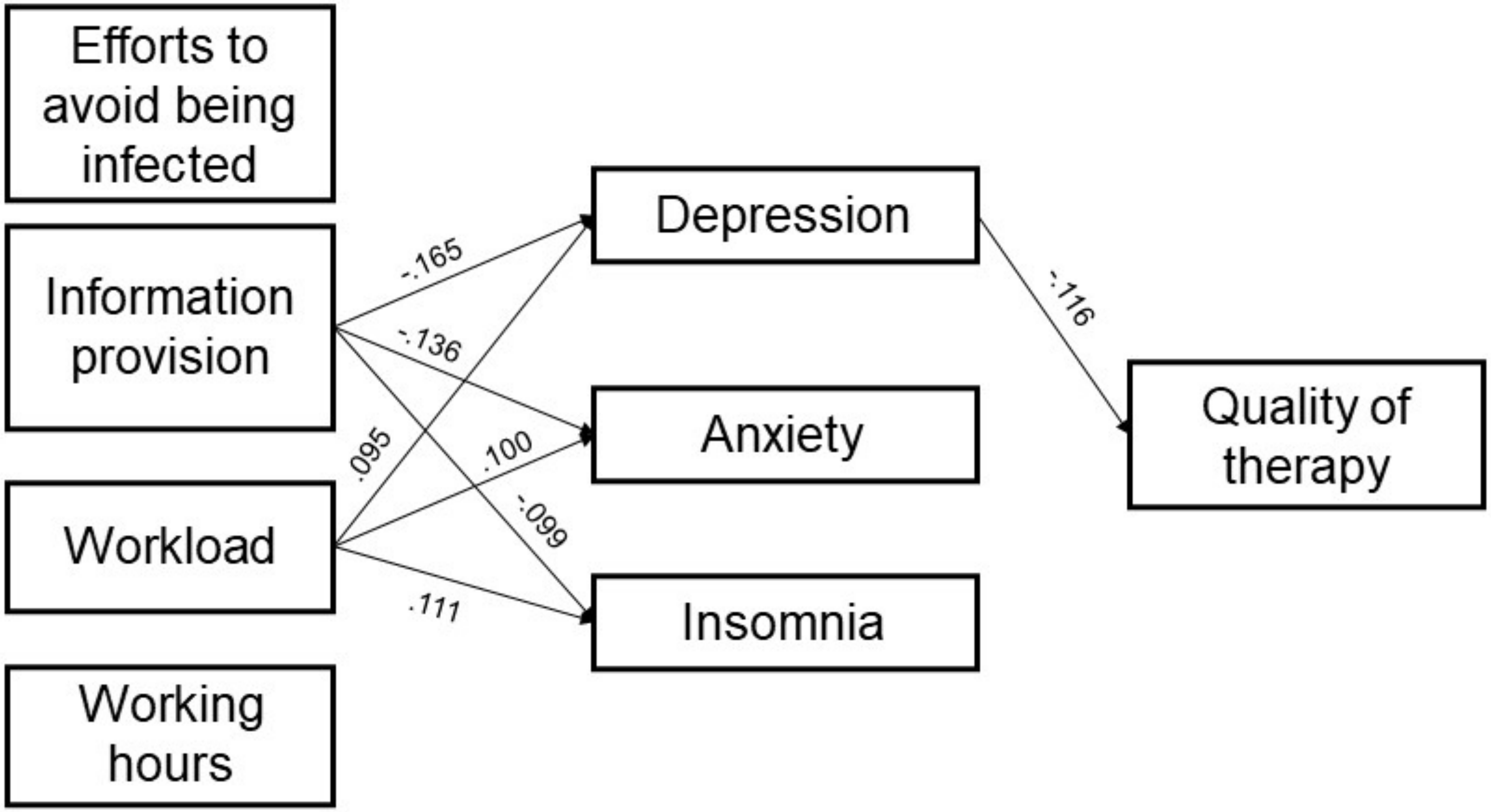

Supplementary Figure 8

Results of the path model for participants who works at hospitals that do not accept patients with COVID-19. Significant paths are depicted with coefficients. For visualization purpose, we retain variables which does not show any significant effects. The results showed group differences in the link between "working hours" and anxiety (Supplementary Figure 7).
